# Fruit Coloration and Anthocyanin Biosynthesis after Bag Removal in Non-Red and Red Apples (*Malus × domestica* Borkh.)

**DOI:** 10.3390/molecules18021549

**Published:** 2013-01-25

**Authors:** Yulian Liu, Fei Che, Lixin Wang, Rui Meng, Xiaojun Zhang, Zhengyang Zhao

**Affiliations:** Department of Pomology, College of Horticulture, Northwest A&F University, Yangling 712100, Shaanxi, China; E-Mails: yulianliu@126.com (Y.L.); aprol_cf@163.com (F.C.); wlxht@nwsuaf.edu.cn (L.W.); yiyan4114@163.com (R.M.); sanshaoye96@nwsuaf.edu.cn (X.Z.)

**Keywords:** anthocyanins, fruit color, PAL, CHI, DFR, UFGT

## Abstract

In the present study, evolution of apple color (L* and a/b), the accumulation of anthocyanins and the activity of the related enzymes, phenylalanine ammonia-lyase (PAL), chalcone isomerase (CHI), dihydroflavonol4-reductase (DFR) and UDP-Glucose: flavonoid-3-*O*-galactosyl transferase (UFGT), were investigated in bagged non-red apple cultivars (‘Granny Smith’ and ‘Golden Delicious’) and red apple cultivars (‘Starkrimon’ and ‘Pink Lady’). Young fruits were bagged 40–45 days after flowering (DAF), and fruits of ‘Golden Delicious’ and ‘Starkrimon’ were uncovered and exposed to light 120 DAF, while those of ‘Granny Smith’ and ‘Pink Lady’ were exposed for 160 DAF. Results showed that cyanidin 3-galactoside (cy3-gal) was the most abundant anthocyanin in both non-red and red cultivars. Level of anthocyanins was higher in ‘Granny Smith’ than in ‘Golden Delicious’, indicating that red color was easier to develop in green cultivar ‘Granny Smith’ than in yellow cultivar ‘Golden Delicious’ after bag removal. The cy3-gal accumulation of non-red cultivars tested was not significantly correlated with PAL, CHI and DFR activity, but was significantly correlated with UFGT activity. During the reddening of non-red apples, UFGT may be the more important factor in the anthocyanin biosynthesis.

## 1. Introduction

Color is the most important indicator of maturity and quality in many fruit species. Anthocyanins represent a group of natural flavonoid compounds in plants and are responsible for coloration [1,2,3]. Over the past decade, studies on cloning of structural genes encoding enzymes in the anthocyanin biosynthetic pathway and the identification of genes encoding transcription factors that regulate the expression of structural genes have been conducted to help better understanding of the molecular basis of anthocyanin biosynthesis in higher plants, such as maize, snapdragon, grapevine, apple and petunia [1,2,4,5,6]. Development of red color of fruits is influenced by genetic factors [6,7,8] and various environmental factors [9,10].

Anthocyanins, condensed tannins, and flavonols are synthesized via the flavonoid pathway, a branch of the phenylpropanoid pathway [11,12,13]. The flavonoid pathway consists of a number of enzymatic steps, each catalyzed by a sequential reaction for flavonoid synthesis [6]. In apple it has been determinated that the accumulation of anthocyanins is majorly correlated to the expression and activity of the enzymes including phenylalanine ammonia-lyase (PAL), chalcone isomerase (CHI), dihydroflavonol4-reductase (DFR) and UDP-Glucose: flavonoid-3-*O*-galactosyl transferase (UFGT) [14,15,16,17]. Environmental factors like temperature and light, water stress and wounding have also proven to be important factors stimulating anthocyanin biosynthesis [18,19,20,21,22,23]. Bagging of fruit can reduce disease and physical damage as well as improve anthocyanin synthesis. This approach has been widely practiced in apple cultivation to produce high quality and unblemished fruit, and to control fruit coloration in China. However, the direct effect of fruit bagging on non-red apple is not fully understood.

About five anthocyanins were identified in apple peel. Cyanidin 3-galactoside (cy3-gal) was identified as the major pigment in apple, accounting for 80% of total anthocyanins [10]. The remaining anthocyanins include cyanidin 3-glucoside (cy3-glu), cyanidin 3-arabinoside (cy3-ara), cyanidin 3-rutinoside (cy3-rut), and cyanidin 3-xyloside (cy3-xyl) [24].

**Figure 1:**
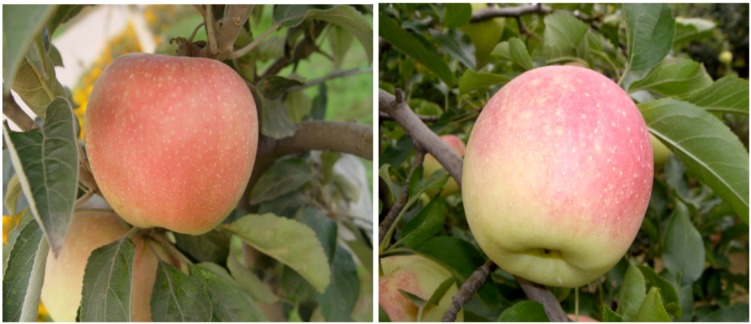
The red ‘Granny Smith’ and ‘Golden Delicious’ (After bag removal, ‘Golden Delicious’ and ‘Granny Smith’ turn red rapidly. At 15 DABR, the surface of the exposed fruit peel developed an intense red color and accumulated anthocyanins).

Anthocyanin biosynthesis in apple fruit was developmentally regulated, with two peaks [3]. The first peak occurrs at the fruitlet stage in both red and non-red cultivars, and the second peak took place later, at the ripening fruit stage in red cultivars only [9]. However, in the arid region of northern Wei River of China, the second peak of anthocyanin accumulation took place at the ripening fruit stage in green cultivar (‘Granny Smith’) and yellow cultivar (‘Golden Delicious’) after bag removal (Figure 1).

The objective of the present study was to investigate color properties and anthocyanin accumulation, and the relationship between activity of related enzymes and anthocyanin accumulation in red and non-red apple.

## 2. Results and Discussion

### 2.1. Color Change

**Figure 2:**
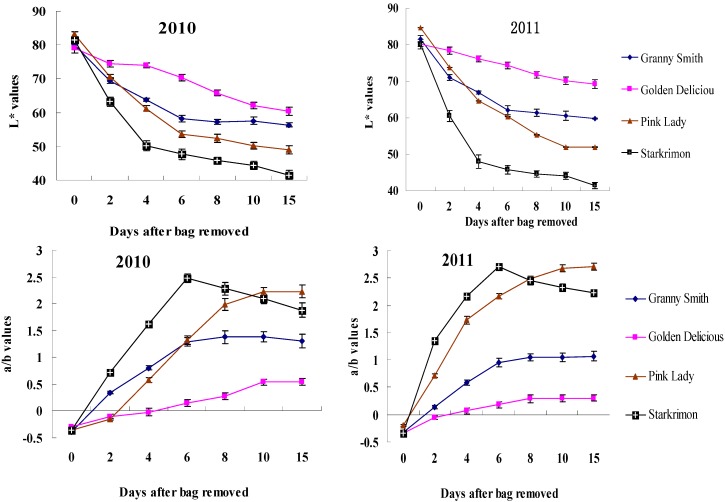
Chormaticity values (L* and a/b) evolution on non-red apple and red apple at different dates after bag removal.

The chromatic characteristics of the fruits of four cultivars during coloring are shown in Figure 2. There were significant differences in L* and a/b between the four cultivars. The change of L* and a/b values between the two kind of cultivars showed similar tendency at different stage of bag removal except ‘Starkrimon’ in 2010 and 2011. At 0 day after bag removal (DABR), all the values of CIE parameter of the non-red cultivars were the same as those of red cultivars. Moreover, the fruits did not develop any red color during they were covered by bags. This showed that bagging had same effects on surface color of the red cultivars and non-red cultivars.

Apple color progressively changed during the first six days after bag removal. The L* values decreased and the a/b values increased with DABR. The results also showed that the first six days after bag removal is a very important stage for the bagged apple coloring, because in this stage, L* and a/b of the four cultivars changed quickly in both years. Interestingly, the a/b values of ‘Starkrimon’ showed a different tendency, and six days after bag removal, the a/b values decreased rapidly, the reason for this is that with coloring and the accumulation of anthocyanins, the surface of ‘Starkrimon’ apples turned dark, which tends to mask the red color. High anthocyanin content may lead to the decrease of fruit brightness. Non-red cultivars after 10 DABR always showed higher L* and lower a/b than those of debagged red cultivars. The L* values of ‘Granny Smith’ and ‘Golden Delicious’ were 56.90 and 60.42 in 2010, and 59.79 and 69.24 in 2011, while those of ‘Pink Lady’ and ‘Starkrimon’ were 49.02 and 41.54 in 2010, and 51.96 and 41.44 in 2011, respectively. Obviously, there are larger differences between the two years in non-red cultivar coloring than in red cultivars.

### 2.2. Anthocyanin Content of Apples

**Figure 3:**
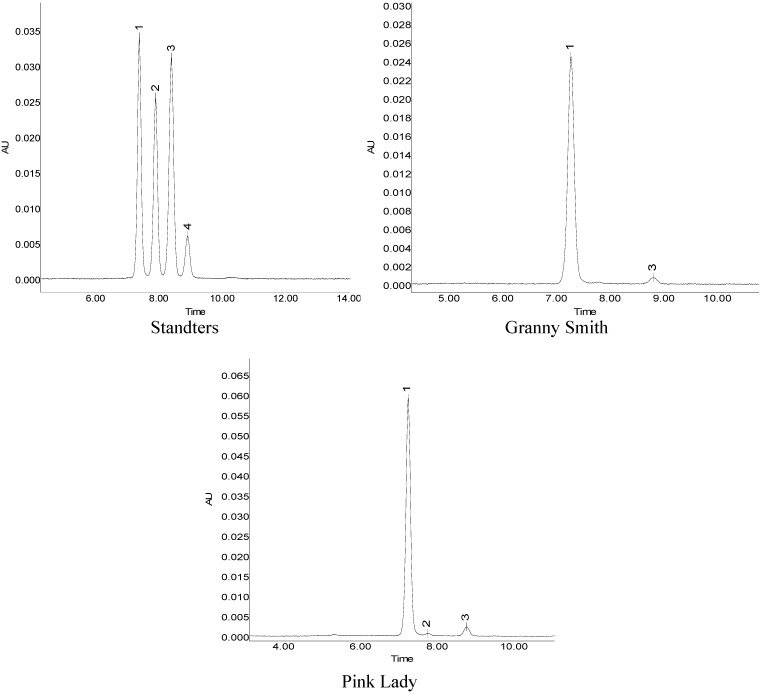
HPLC separation of anthocyanins in apple cultivars monitored at 530 nm.Peaks: (1) cyanidin 3-galcoside; (2) cyanidin 3-glucoside; (3) cyanidin 3-arabinoside; (4) cyanidin 3-rutinoside.

**Figure 4:**
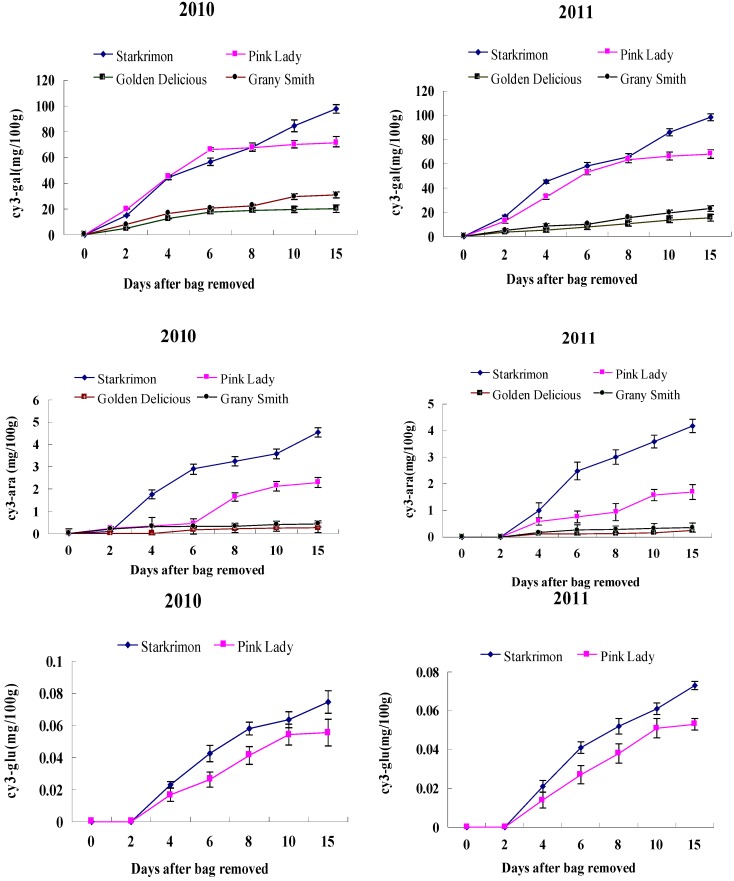
Anthocyanins (cy3-gal, cy3-ara and cy3-glu) of non-red apple and red apples at different dates after bag removal.

Analysis of anthocyanin composition and concentration in the skin samples of the coloring fruits collected at different times was carried out by HPLC. The HPLC chromatograms of apple extracts obtained at 530 nm revealed three peaks (Figure 3), which corresponded to the three anthocyanins cy3-gal (peak 1), cy3-glu, (peak 2) and cy3-ara (peak 3). After bagging treatment, ‘Granny Smith’ (green cultivar) and ‘Golden Delicious’ (yellow cultivar) gradually turn red. The red blush on fruit was significantly increased by bagging fruit, irrespective of year and cultivars. Two anthocyanins, cy3-gal and cy3-ara, were identified in ‘Granny Smith’, and ‘Golden Delicious’. Cy3-gal, chiefly responsible for their red color, ranged from 0 to 29.56 mg/100 g FW in ‘Granny Smith’ and 0 to 19.59 mg/100 g FW in ‘Golden delicious’ in 2010, and from 0 to 23.04 mg/100 g FW in ‘Granny Smith’ and 0 to 15.35 mg/100 g FW in ‘Golden Delicious’ in 2011. Cy3-ara was present in smaller amounts in the reddening of two cultivars, Cy3-glu was not detected in non-red cultivars (Figure 4). In red cultivars, cy3-gal was the major anthocyanin, which ranged from 0 to 71.41 mg/100 g FW in ‘Pink Lady’ and 0 to 97.84 mg/100 g FW in ‘Starkrimon’ in 2010. In 2011, it varied from 0 to 68.09 mg/100 g FW in ‘Pink Lady’ and 0 to 98.48 mg/100 g FW in ‘Starkrimon’, followed by cy3-ara and cy3-glu. Although the non-red cultivars turn red after bag removal, the concentrations of anthocyanins in the skin were significantly lower than in red cultivars. Concentrations of cy3-gal (the major anthocyanin) in red cultivars were about 3-fold higher than observed in non-red cultivars.

In previous studies, cy3-gal was found to be the most abundant anthocyanin in red cultivars [20,25]. In this study, we found that cy3-gal was the most abundant anthocyanin in both non-red and red cultivars, accounting for more than 94% of the total anthocyanins. According to the earlier studies, other anthocyanins which presented in minor amounts in some apple cultivars [10], such as cy3-rut, cyanidin 3, 5-diglucoside, and cyanidin 3-acetylglucoside, were not detected. It was possibly due to the quite low total anthocyanin content in these cultivars. Generally, the same anthocyanins were present in each apple cultivar, but were at different relative levels for different cultivars. Cy3-gal predominantly accumulated in the ripe apple fruit skin regardless of the time (DABR) or cultivars. Similar results were obtained in previous studies [24,25,26]. Previously, the phenomenon that the non-red cultivars turn to red were reported [9,26,27], but for kinds and contents of anthocyanins in non-red cultivars were rarely reported in papers.

When bags were removed from ‘Granny Smith’ and ‘Golden Delicious’ fruit, substantial increases development of red blush on the surface, and the higher content of anthocyanins were measured in ‘Granny Smith’ than in ‘Golden Delicious’, indicating that green cultivar ‘Granny Smith’ turned red easier than the yellow cultivar ‘Golden Delicious’ after bag removal under the same climatic conditions. The exception was ‘Granny Smith’ which had 5-fold higher levels of MdMYB1 transcripts than ‘Golden Delicious’, and had higher levels of structural gene (CHS, DFR and UFGT) transcripts than ‘Golden Delicious’ after bag removal [6]. These may be the reasons for the fact that ‘Granny Smith’ was turned red easier than the yellow cultivar ‘Golden Delicious’. 

### 2.3. Activity of PAL, CHI, DFR, UFGT

It has been known that the biosynthesis of anthocyanins in maturing fruit is a light-dependent process, and light of sufficient energy and spectral composition is necessary to initiate anthocyanin synthesis [3,10,20]. In this study, bagging treatment inhibited the biosynthesis of anthocyanin in all cultivars as no anthocyanins were detected in any of the cultivars at 0 DABR and activities of the four enzymes were strongly inhibited. In previous study, bagging has been observed to be one of the main strategies for improving fruit coloration in apple [28,29]. After bagging, formation of anthocyanins was greatly reduced due to the blockage of anthocyanin biosynthesis gene expression and activity of related anthocyanin biosynthesis enzymes [28,30]. After bag removal, anthocyanin biosynthesis genes were coordinately expressed rapidly and the activity of related anthocyanin biosynthesis enzymes increased obviously [2,27].

**Figure 5:**
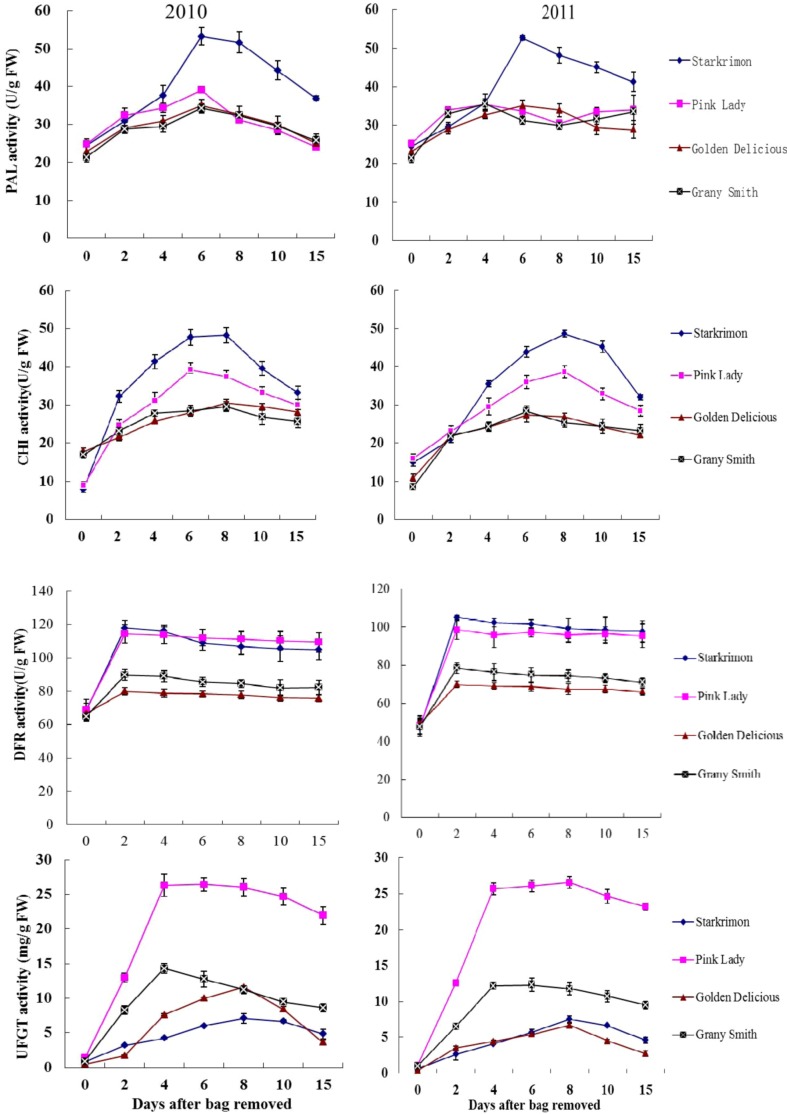
Enzyme activities of PAL, CHI, DFR and UFGT from the peel of ‘Granny Smith’, ‘Golden Delicious’, ‘Pink Lady’ and ‘Starkrimon’ after bag remvoal.

Anthocyanin synthesis is a process that involves many steps [3]. PAL, CHI, CHS, F3H, DFR, ANS and UFGT are closely related with anthocyanin biosynthesis in apple [14,15,16,17]. After bag removal, levels of the four enzymes PAL, CHI, DFR and UFGT were used to examine the patterns of change of anthocyanin biosynthesis (Figure 5). PAL was the first enzyme to catalyse the elimination of NH_3_ from L-phenylalanine to give *trans*-cinnamate [10]. PAL activity has been reported to positively correlate with anthocyanin synthesis in grapes, strawberries and apples, but its role in regulating anthocyanin formation in apples remains controversial [28,31,32,33,34]. In 2010 and 2011, the PAL activity in all apple fruits increased during 0–6 DABR, then decreased. Wang *et al.* also found the PAL activity of bagged ‘Jonathan’ increased up to 96 h under light irradiation, then decreased after 96 h [14]. At 0 DABR, the PAL activity of four cultivars was 21.47–25.23 U/g FW. After bag removal, the PAL activity of four cultivars ‘Starkrimon’, ‘Golden Delicious’, ‘Granny Smith’ and ‘Pink Lady’ increased rapidly. The PAL activity of four cultivars were 31.23–56.25 U/g FW at 4–6 DABR. PAL activity decreased at 15 DABR, and was also higher than that of apple fruit at 0 DABR. The changing trend of PAL activity in ‘Golden Delicious’ and ‘Starkrimon’ fruit was same in both 2010 and 2011. But the PAL activity in ‘Granny Smith’ and ‘Pink Lady’ was different, as it showed a gradual increasing trend after 8 DABR. During 4–15 DABR, PAL activity in ‘Starkrimon’ was more than two times as high as that of other three cultivars.

In this study, only the PAL activity of ‘Starkrimon’ was generally higher than the other three cultivars after bag removal. It is well known that ‘Starkrimon’ is a dark red cultivar, and a higher content of anthocyanins was measured in ‘Starkrimon’ (Figure 4), hence PAL activity may have a correlation with the accumulation of anthocyanins. The anthocyanin accumulation was not correlated with PAL activity in non-red and red cultivars (Table 1). After 8 DABR, the PAL activity showed a gradual increase in ‘Granny Smith’ and ‘Pink Lady’, meanwhile, the anthocyanin accumulation of the two cultivars remained unchanged. This indicated that the change of PAL activity was not consistent with anthocyanin accumulation. Wang *et al.* [14] concluded that PAL was not the only regulating factor for anthocyanin accumulation in bagged mature and ripe fruit. Conclusively, PAL activity was important for anthocyanin synthesis, but not the only regulating factor for anthocyanin accumulation in bagged fruit.

**Table 1:** Correlation coefficients between cy3-gal with PAL, CHI, DFR and UFGT of apple reddening.

Cultivar	year	anthocyanin	PAL	CHI	DFR	UFGT
Granny Smith	2010	cy3-gal	0.453	0.339	0.436	0.881 *
	2011		0.625	0.570	0.450	0.814 *
1	2010	cy3-gal	0.529	0.615	0.271	0.845*
	2011		0.366	0.614	0.496	0.884*
Pink Lady	2010	cy3-gal	0.207	0.793 *	0.684	0.901 **
	2011		0.365	0.858 *	0.644	0.856 *
Starkrimon	2010	cy3-gal	0.577	0.764 *	0.401	0.865 *
	2011		0.631	0.827 *	0.562	0.821 *

Values are means ± SE (n = 4); ** indicates *p* < 0.01, * indicates *p* < 0.05.

CHI plays an important role in the transformation process of flavonoid compounds; transformation by the stereospecific action of CHI provides the first flavonoid a (2*S*)-flavanone (naringenin) [35]. At 0 DABR, The CHI activity of four cultivars was 7.96–14.83 U/g FW. After bag removal, the activity of CHI tended to rise, then decreased, and peaked at 6–8 DABR, The CHI activity of four cultivars were 23.53–49.28 U/g FW. To 15 DABR, CHI activity decreased, and was also higher than that of apple fruit at 0 DABR. It appeared that there was a good correlation between CHI activity and red cultivars. This is consistent with previous findings in apple and red pear [15,36,37]. However, after bag removal, the CHI activity of non-red apple fruit did not change significantly, and there was no correlation between the CHI activity and cy3-gla. Takos *et al.* [6] found the *MdCHI* gene was the only anthocyanin pathway gene that did not seem to have reduced expression in non-red peel apple cultivars at fruit ripening. In this study, ‘Granny Smith’ and ‘Golden Delicious’ cannot turn red under natural light, but when fruits treated with two-layered bags were exposed to light, they started to accumulate anthocyanin rapidly. Furthermore, lower content of anthocyanins were measured in ‘Granny Smith’ and ‘Golden Delicious’ than in ‘Starkrimon’ and ‘Pink Lady’ (Figure 4). These observations indicate only when high content level of anthocyanins in apple peel, it appeared that there was a good correlation between CHI activity and anthocyanins accumulation.

At 0 DABR, the DFR activities of all cultivars did not show any significant differences. During 0–2 DABR, the DFR activity increased rapidly, then retained unchanged till 15 DABR in both years. In addition, the DFR activities were higher in 2010 than in 2011, and higher activity was seen in red cultivars than non-red cultivars. Ju *et al.* [16] found that DFR activity was higher in the red fruited cultivars than that in non-red fruit cultivars. Similar results were also obtained in our study. The important point for anthocyanin synthesis in apple was suggested to be located between the reactions from dihydroquercetin to cyanidin formation [15]. Han *et al.* [38] reported ectopic expression of apple ANR genes in tobacco flowers inhibits expression of DFR genes, resulting in loss of anthocyanins. According to these results we conclude that DFR is necessary, but not a limiting point, for anthocyanin synthesis in apples.

UFGT activity of ‘Pink Lady’ and ‘Granny Smith’ peaked at 4 DABR, decreased from 4 to 15 DABR in both 2010 and 2011. The UFGT activity peaked at 8 DABR, and decreased from 8 to 15 DABR in ‘Starkrimon’ and ‘Golden Delicious’ in both years. Activity of UFGT was generally higher for ‘Pink Lady’ and ‘Granny Smith’, and was not different from the other two cultivars. Highly significant correlation values were observed between the UFGT activity and cy3-gal, in red cultivars and no relation to synthesis of anthocyanins in ‘Granny Smith’ and ‘Golden Delicious’, the correlations being higher than 8.14. In apple skin, the higher abundance of cy3-gal as compared to cy3-glu suggests that the functional enzyme is UFGT, which is responsible for the transfer of galactose to the 3-O-position of anthocyanins [17]. UFGT have already been thought to be the most important enzyme in the development of red pigmentation for apple and litchi [4,5,16,38]. A highly significant correlation between the UFGT activity and cy3-gal was observed for all cultivars. During the reddening of non-red apples, there was a good correlation between UFGT and cy3-gal, hence we can conclude that UFGT may be the most important factor in the biosynthesis of anthocyanins.

Anthocyanin accumulation is thought to be the result of interaction of the multiple key enzymes in the flavonoid pathway [28]. In this study, bagging treatment inhibited the biosynthesis of anthocyanins in all cultivars as no anthocyanins were detected in all cultivars at 0 DABR and activities of four enzymes were strongly inhibited. Five genes (CHS, F3H, DFR, ANS and UFGT) were detected in the exposed portion of non-red ‘Mutsu’ apple skin after bag removal [27], so the anthocyanins formed after bagging of non-red cultivars are identical to those occurring normally in red cultivars.

## 3. Experimental

### 3.1. Plant Materials

Five-six years old apple plants were used in the present study, grown on the Apple Experimental Farm of Northwest A&F University (35°21′N, 109°30′W; elevation 850 m; Shaanxi, China). Its climate corresponds to the warm temperate zone (average annual temperature 11.4 °C, average annual rainfall 577.8 mm). All apple cultivars were grafted on M26 rootstock and planted in 4 × 2 m density. Young fruits were bagged 40 days after flowering (DAF) with a two-layer paper bag (Hong Tai, Xi’an, China), the size of the bag is 13 × 16 cm and it is made of red paper, with its inner layer coated with wax. Bagged fruits of ‘Golden Delicious’ and ‘Starkrimon’ were exposed to light 120 DAF, while those of ‘Granny Smith’ and ‘Pink Lady’ 160 DAF. Fruits were harvested after 0, 2, 4, 6, 8, 10 and 15 day of bag removal, respectively, and used for various experiments. For color measurement of fruit peel, each treatment was replicated four times with four apples randomly selected from among 10 fruits. Enzyme and anthocyanin concentration measurements were carried by ultraviolet spectrophotometer and HPLC. After color measurements, peels were collected in plastic bags, frozen at −20 °C until used for measurements of enzymes and anthocyanins.

### 3.2. Fruit Color Measurement

Apple color was measured using the CHROMA METER CR-400 Chromaportable colorimeter (Konica Minolta Sensing, inc., Osaka, Japan). Fruit chromaticity was recorded in CIE parameters L*, a* and b* color space coordinates. The colorimeter was calibrated with a white standard calibration plate before use. L* value represented the relative lightness of colors ranging from 0 (black) to 100 (white). Values of a* and b* ranged from −60 to 60, where a* was negative for green color and positive for red color, and b* was negative for blue and positive for yellow [39].

### 3.3. Determination of Enzyme Activity

#### 3.3.1. PAL and CHI Enzyme Extraction

Fruit peel (0.50 g) was ground to a fine powder with a mortar containing liquid nitrogen. Extraction of PAL and CHI enzymes was performed according to Lister and Lancaster [15]. Briefly, the powder was extracted with 50 mM phosphate buffer (250 mL, pH 7.0) containing 5% (w/v) PVP, 50 mM Na ascorbate, 18 mM mercaptoethanol and 0.1% Triton X-100. Then the extracts were filtered and centrifuged at 20,000 ×*g* for 20 min. Ammonium sulphate was added to the supernatant to obtain 35% saturation. The supernatant was centrifuged for 20 min at 20,000 ×*g* to remove the PVP, and then more ammonium sulphate was added to reach a final saturation of 80%. The homogenate was centrifuged at 20,000 ×*g* for 20 min and the pellet resuspended in 1–2 mL extraction buffer (without PVP and Triton) and incubated overnight to obtain purified extracts, which were used for enzyme analysis. All procedures were performed at 4 °C.

#### 3.3.2. Assay of PAL Activity

PAL was assayed as described by Lister and Lancaster [15]. The assay mixture consisted of 50 mM borate buffer (2.5 mL) and crude enzyme (0.5 mL) and the reaction initiated by the addition of L-phenylalanine. Tubes were incubated at 34 °C for 30 min and the reaction was stopped by addition of 35% (w/v) trifluoroacetic acid (0.5 mL), followed by centrifugation for 10 min at 5,000 ×*g* to get the pellet of the denatured protein. The yield of cinnamic acid was estimated by measuring A_290_ of the supernatant in 10 mm quartz cuvettes.

#### 3.3.3. Assay of CHI Activity

CHI activity was determined according to Lister and Lancaster [15]. Partially purified enzyme (500 µL) was added to 50 mM Tris-HCl (pH 7.4) containing BSA (at a final concentration of 7.5 mg·mL^−1^) and 50 mM KCN (to inhibit peroxidase degradation of the tetrahydroxychalcone) to give a final volume of 2.5 mL. Tubes were incubated at 34 °C for 30 min. To allow for spontaneous isomerisation of the chalcone the reference cell contained the assay mixture without enzyme. The reaction was stopped by heating to boiling for 10 min. The initial rate of disappearance of the chalcone (ΔA_381_), in the presence of enzyme, was used to estimate CHI activity.

#### 3.3.4. DFR and UFGT Enzyme Extraction

UFGT and DFR were extracted according to Fukuchi-Mizutani *et al.* [36]. The frozen apple peels samples (1.00 g) were ground in liquid nitrogen, and the powdered tissues were suspended in buffer A [6 mL, 100 mM Tris-HCl, pH 7.5; 10 mM sodium ascorbate; 5 mM dithiothreitol, 0.1% β-mercaptoethanol and 10 µM *p*-aminophenyl methanesulfonyl fluoride (*p*-APMSF)]. After centrifugation at 12,000 ×*g* for 20 min, the supernatant was fractionated with ammonium sulfate to between 40% and 70% saturation. The precipitated protein was dissolved in a minimum volume of buffer B (20 mM Tris-HCl, pH 7.5; 0.1% [v/v] β-mercaptoethanol; 10% [v/v] glycerol and 10 µM *p*-APMSF). All of the procedures described above were performed at 4 °C.

#### 3.3.5. Assay of DFR Activity

DFR activity was measured according to Stafford [35]. Incubation mixture (1.1 mL) contained 0.1 M Tris-HCl buffer (pH 7.4), 1 µmol each of (+)-dihydroquercetin and NADPH, and a regenerating system for NADPH consisting of 6 µmol of glucose-6-P and 1 unit of glucose-6-P dehydrogenase. After incubation at 30 °C for 1 h, 15 µL of 6 N HCl was added and the mixture was immediately extracted with 3 × 1 mL of ethyl acetate; the latter was backwashed with 3 × 0.2 mL of H_2_O. The ethylacetate was evaporated with a vacuum at 35 °C to 40 °C into a 0.1 mL aqueous phase. The 3,4-diol content was determined by the production of cyanidin at 95 °C after the addition of 1 mL butanol-HCl reagent. The absorbance due to cyanidin was measured at 550 nm.

#### 3.3.6. Assay of UFGT Activity

UFGT activity was measured using quercetin and UDP-Galactose as substrates [17]. A standard reaction mixture consisted of 100 µL (250 µM) quercetin, 70 µL (500 µM) UDP-Galactose, and 200 µL of enzyme solution in 200 µL of 0.1 M potassium phosphate buffer (pH 8.5). The mixture was incubated for 30 min at 30 °C, and the reaction was terminated by adding 130 µL 30% (v/v) trichloroacetic acid. After centrifugation at 12,000 ×g for 10 min, the supernatant was analyzed by HPLC equipped with a reverse phase column Diamonsil C18 (L) 5 μ (250 × 4.6 mm, Dikma, Beijing, China).

### 3.4. Extraction, Purification, and Isolation of Anthocyanins

For high-performance liquid chromatography (HPLC) analysis, anthocyanins were extracted from 1.00 g finely ground plant material in 1 mL 1% (v/v) HCl-methanol for 24 h at 4 °C temperature on a rotating wheel in the darkness. Samples were clarified by centrifugation at 13,000 ×*g* for 15 min at 4 °C and then 1.5 mL of supernatant was transferred to autosampler vials. Analysis was conducted with the HPLC PDA (Waters, Milford, MA, USA), which was equipped with a model 1525 binary solvent delivery system (Waters), an on-line degasser, and 2707 autosampler (Waters). The data acquisition and processing were performed by the Breeze software. For all samples, anthocyanin separations were carried out on a 250 × 4.6 mm i.d., 5 μm C18 column (Diamonsil). Solvent A was 10% (v/v) formic acid and solvent B was methanol. The gradient of solvent B was: 0 min, 17%; 1 min, 17%; 10 min, 35%; 20 min, 37%; 25 min, 100%. The gradient was run at a flow rate of 1 mL·min^−1^, at a column temperature of 40 °C and a 5 μL sample was injected. Absorbance was measured at 520 nm. Standards were Cy3-gal, cy3-glu (Sigma Chemical, St. Louis, MO, USA), cy3-ara and cyaniding-3-rutinoside chloride (Cy3-rut) (Polyphenols Laboratories AS, Hanaveien, Norway).

### 3.5. Statistical Analysis

Experimental data from four cultivars were statistically processed using SPSS version 17.0 (SPSS inc., Chicago, IL, USA). The Pearson’s correlations were performed on anthocyanin and enzymes activity’s original data.

## 4. Conclusions

When dark-grown ‘Granny Smith’ and ‘Golden Delicious’ were exposed to sunlight, fruits rapidly turned red. The L* values decreased and a/b values increased with the apple coloring. Non-red apples had higher L* values than those of red apples. The two constituents responsible for apple red peel color were cy3-gal and cy3-ara, and the most abundant anthocyanin in non-red cultivars was cy3-gal. Green cultivar ‘Granny Smith’ turned red more easily than the yellow cultivar ‘Golden Delicious’ after bag removal. During the non-red apples reddening, UFGT may be the more important factor in the biosynthesis of anthocyanins.
